# Robotic Management of Complex Obstructive Megaureter Needing Ureteral Dismembering and/or Tapering in Children: A Single-Center Case Series

**DOI:** 10.3390/medicina60111837

**Published:** 2024-11-08

**Authors:** Ciro Esposito, Lorenzo Masieri, Francesca Carraturo, Annalisa Chiodi, Claudia Di Mento, Giorgia Esposito, Mauro Porcaro, Daniella Araiza Kelly, Maria Escolino

**Affiliations:** 1 Division of Pediatric Surgery, Federico II University Hospital, 80131 Naples, Italy; ciroespo@unina.it (C.E.); francesca.carraturo@unina.it (F.C.); annalisa.chiodi@unina.it (A.C.); claudia.dimento@unina.it (C.D.M.); pedsurg.esposito@unina.it (G.E.); 2 Division of Pediatric Urology, Meyer Children University Hospital, 50139 Florence, Italy; 3 Division of Pediatric Radiology, Federico II University Hospital, 80131 Naples, Italy; 4 Division of Pediatric Surgery, Anáhuac University, Mexico City 52786, Mexico

**Keywords:** obstructive megaureter, complex, anatomy, reimplantation, robot, children

## Abstract

*Background and Objectives*: Robot-assisted extravesical ureteral reimplantation (REVUR) has been described as valuable alternative to open reimplantation in the pediatric population. This study aimed to report the outcome of REVUR in children with complex obstructed megaureter (COM) needing ureteral dismembering and/or tapering. *Materials and Methods*: The records of patients with COM, who received REVUR with ureteral dismembering and/or tapering over the last 3 years (2021–2024), were retrospectively reviewed. The inclusion criteria for COM included previous surgery, paraureteral diverticula, or ectopic megaureter. *Results*: A total of 16 patients (15 boys), with a median age of 7.8 years (range 2–16), were treated over the study period. COM was associated with paraureteral diverticula (*n* = 6), previous failed endoscopic balloon dilation (*n* = 4), ectopic megaureter (*n* = 2), and previous bulking agent endoscopic injection causing iatrogenic ureteral obstruction (*n* = 4). Presentation symptoms included febrile urinary tract infections (*n* = 8), flank pain (*n* = 4), hematuria (*n* = 2), and pseudo-incontinence (*n* = 2). All surgical procedures were accomplished robotically without conversions or intra-operative complications. Ureteral tapering was performed in 7/16 (43.7%). The median operative time (including robot docking) was 220 min (range 155–290). The median length of stay was 3.8 days (range 3–7). The indwelling double J stent was removed 4–6 weeks postoperatively. Clavien 2 grade complications occurred postoperatively in 2/16 (12.5%). At median follow-up of 34.5 months, all patients were asymptomatic and showed improved hydroureteronephrosis on ultrasound and improved drainage on diuretic renogram. *Conclusions*: This study demonstrates that robot-assisted extravesical ureteral reimplantation is a safe and effective treatment for primary obstructive megaureter and other complex ureteral anomalies in our patient cohort. The procedure showed low complication rates, high success rates, and favorable long-term outcomes, supporting the feasibility and effectiveness of robotic surgery for these conditions.

## 1. Introduction

The uretero-vesical junction (UVJ) plays a crucial role in maintaining the proper function of the urinary tract by ensuring the unidirectional flow of urine from the kidneys to the bladder. However, congenital or acquired abnormalities at this junction, such as primary obstructive megaureter (POM) and vesicoureteral reflux (VUR), can lead to significant clinical consequences, including recurrent infections, hydronephrosis, and potential renal damage [1,2,3]. Megaureter is defined as a dilation of the ureter with a diameter greater than 7 mm, regardless of the underlying cause. The proper classification of each case as refluxing, nonrefluxing, obstructed, or nonobstructed is crucial and is typically performed through diagnostic tests such as ultrasound, voiding cystourethrogram (VCUG), nuclear medicine drainage studies, and/or magnetic resonance urography [1]. There has been a growing preference for the conservative management of megaureters, as most cases tend to resolve or improve over time. However, in rare situations where hydroureteronephrosis (HUN) does not improve, is linked to urinary tract infections (UTIs), or shows signs of obstruction with reduced kidney function, surgical intervention becomes necessary [2,3].

Historically, this condition has been managed through open surgical techniques, but the advent of minimally invasive methods has revolutionized the surgical approach to this pathology [4,5,6]. In recent years, endoscopic balloon dilation has offered a minimally invasive alternative to ureteral reimplantation (UR) for children with persistent or progressive POM, despite a modest long-term success rate [7]. In the case of failure of endoscopic treatment, surgical reimplantation is indicated. The most recent EAU/ESPU guidelines on VUR recognize both laparoscopic and robotic methods as viable options, offering comparable resolution and complication rates [8].

Robotic-assisted surgery has demonstrated significant effectiveness in pediatric urology, with outcomes that may be comparable to those of traditional open or laparoscopic methods [9]. Robotic surgery, with its enhanced precision, reduced postoperative pain, and faster recovery times, is increasingly being employed for complex urological procedures in children [10]. Robot-assisted extravesical ureteral reimplantation (REVUR) has been increasingly adopted as a valuable alternative to open reimplantation in the pediatric population [11,12,13,14,15,16]. The robotic approach may also be appropriate for patients with more complex ureteral and bladder anomalies at the UVJ [17,18,19]. 

A robotic-assisted technique addresses some of the technical difficulties, particularly in laparoscopic suturing, and, therefore, may lead to wider adoption when adequate resources are available. This shift in surgical practice is supported by a growing body of evidence, although the long-term outcomes and comparative effectiveness of robotic versus traditional methods continue to be areas of active research [20,21,22]. 

As the field continues to evolve, large-scale studies and long-term follow-up will become essential to fully establish the role of robotics in pediatric urology and refine the techniques that will define the future standard of care. We hypothesized that robotic surgery is a safe and effective method for performing complex lower urinary tract reconstructive procedures across all age groups, especially in patients with difficult anatomical conditions. 

This study aimed to report a single-center experience about the outcome of REVUR in children with complex obstructive megaureter (COM) needing ureteral dismembering and/or tapering. 

## 2. Materials and Methods

### 2.1. Patient Selection

We conducted a retrospective cohort study of all patients with complex obstructed megaureter (COM) who underwent robot-assisted extravesical ureteral reimplantation (REVUR) and ureteral dismembering and/or tapering over a 5-year period (January 2019 to January 2024). Patient data were sourced from a robotic surgery registry stored in a Research Electronic Data Capture database.

Eligibility for inclusion required a primary diagnosis of obstructed megaureter associated with complex characteristics such as previous surgery on the ipsilateral UVJ, paraureteral diverticulum (PUD), and ectopic megaureter. 

Exclusion criteria were primary VUR without evidence of obstruction, bladder exstrophy and neurogenic bladder, or concurrent anomalies such as ureteropelvic junction obstruction. Patients weighing less than 10 kg were also excluded from the robotic approach in our practice. 

### 2.2. Operative Technique

All surgeries were performed by 2 pediatric urologists at a single institution, who had fellowship training, including experience with robotic surgery. All procedures were conducted using the Da Vinci Xi system with 8 mm robotic instruments.

Patient Setup: The procedure begins with general anesthesia, orotracheal intubation, and muscle relaxation. The patient is placed in a supine position on the operating table with a slight Trendelenburg tilt. A sterile catheter of appropriate size is inserted into the bladder before starting the surgery and is used to manage bladder filling and emptying during the procedure.

Port Placement: Three 8 mm robotic ports and one 5 mm assistant port are placed. The robotic system is then docked at the patient’s feet in the pelvic position.

**Figure 1:**
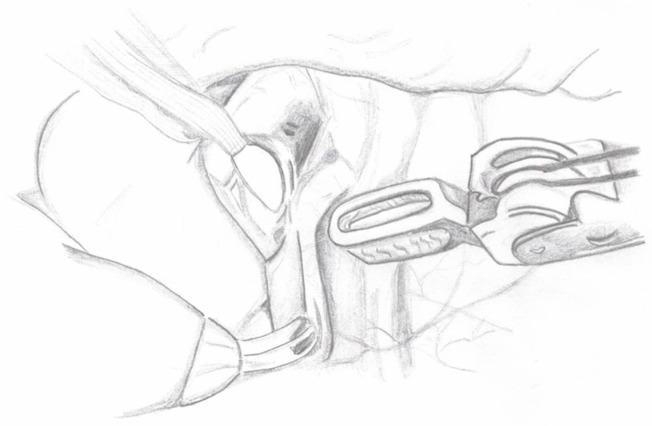
Ureter dissection.

**Figure 2:**
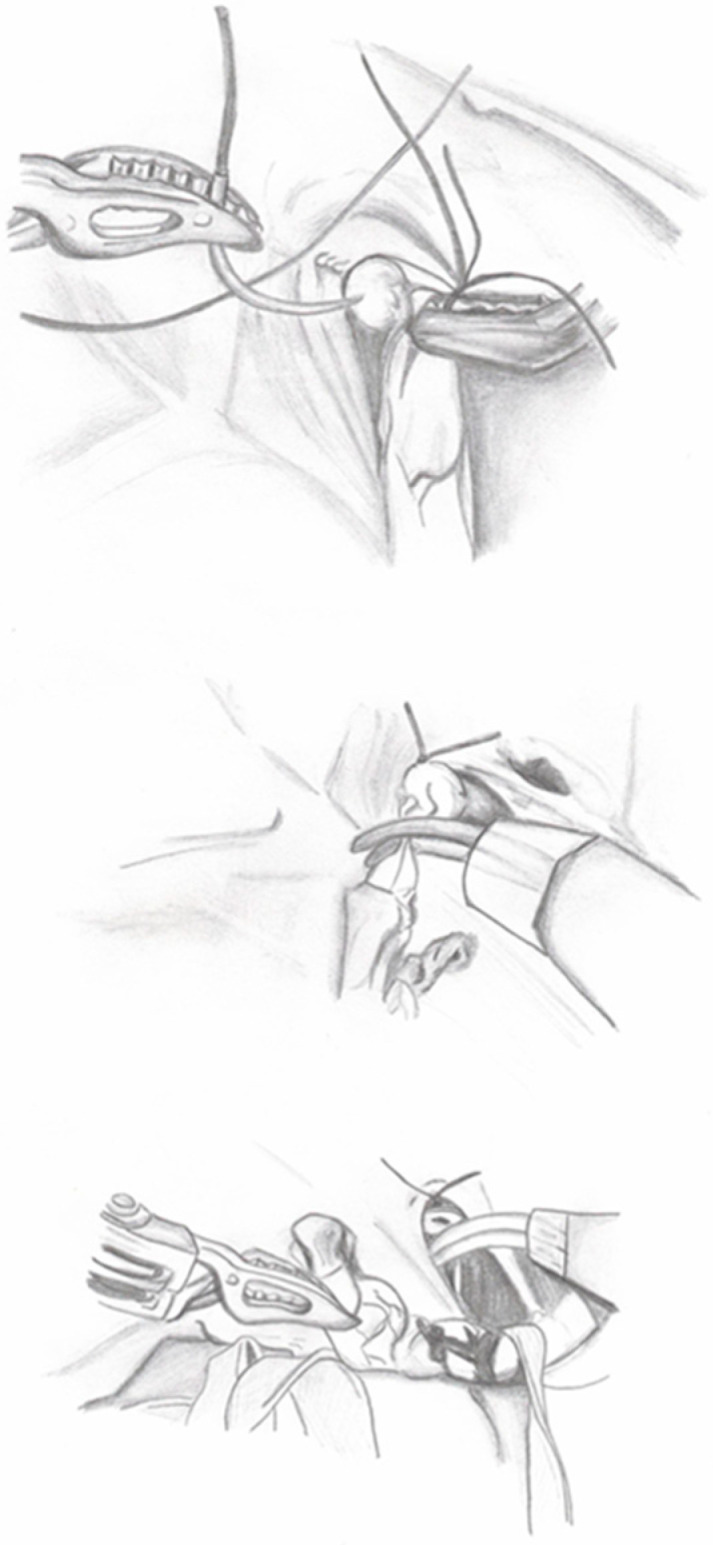
Ureter dismembering.

**Figure 3:**
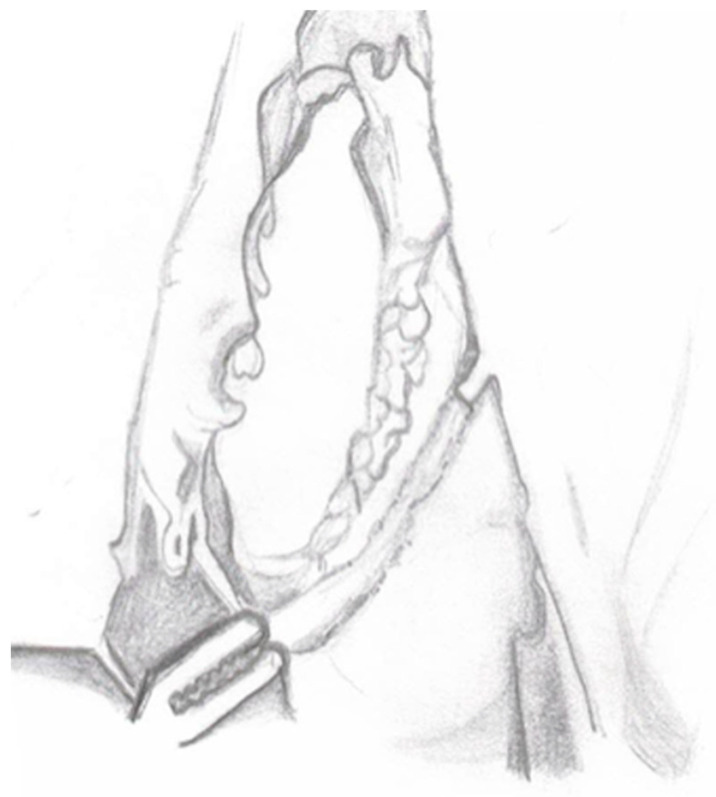
Detrusorotomy.

**Figure 4:**
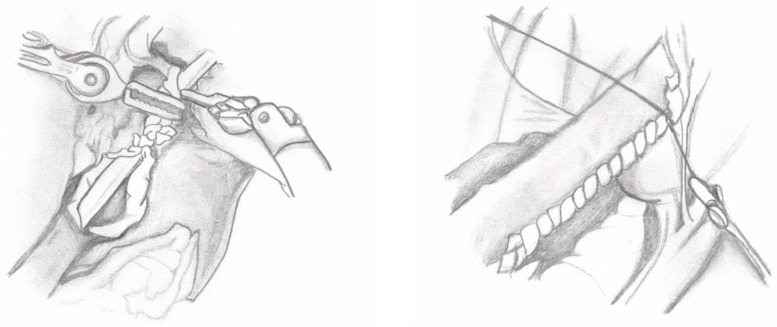
Ureter tapering.

**Figure 5:**
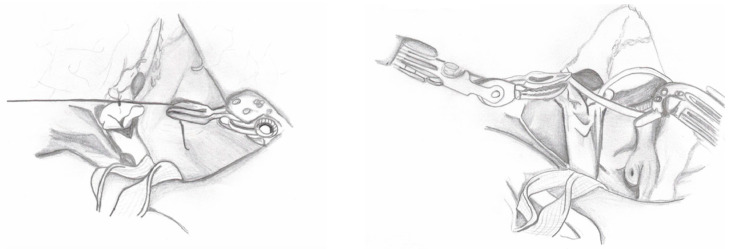
Ureteroneocystostomy and double-J stent placement.

**Figure 6:**
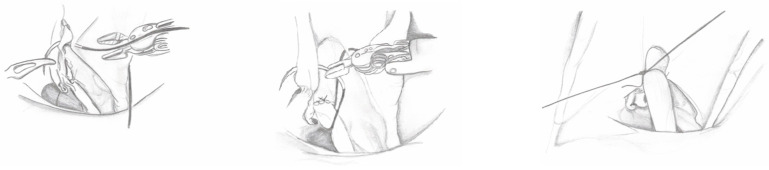
First stitch of ureteral extravesical reimplantation.

Surgical Steps:

1. Ureter Dissection: The peritoneum is incised below the iliac bifurcation to expose the ureter. In females, the space between the uterus and bladder is identified; in males, the posterior peritoneum of the bladder is incised and reflected to visualize the ureter. Dissection is carried out carefully to avoid nerve damage, with attention to surrounding vessels and minimal use of electrocautery. A cotton tape is placed around the ureter to minimize manipulation. Dissection proceeds distally below the vessels in males, below the uterine artery in females, and around the ureterovesical junction (UVJ) (Figure 1).

2. Creation of Peritoneal Window: A peritoneal window is created behind the large uterine ligament in females or the vas deferens in males to allow for the passage of the ureter.
3. Bladder Preparation: Transabdominal stitches are used to lift the bladder.
4. Ureteral Dismembering: The distal ureter is ligated with a 2-0 absorbable suture and separated from the bladder (Figure 2). In the case of PUD, the ureter is dissected until the point where it appears to enter the diverticulum. Thereafter, the diverticulum is ligated and excised.

5. Detrusorotomy: The bladder is filled with saline to facilitate detrusorotomy. The detrusor tunnel is marked from the UVJ to about 5 cm on the bladder’s back wall using monopolar scissors. The detrusor muscle is cut in layers until the mucosa is visible, forming valves for the ureter to tunnel through (Figure 3).

6. Ureteral Tapering: The excess ureter is removed, and a neo-hiatus is created using an absorbable suture to maintain tension. The ureter is then tapered and sutured with simple monofilament sutures (Figure 4). The decision for ureteral tapering is approached intraoperatively with great caution. After dismembering, the ureteral diameter is measured, and tapering continues only if the ureteral diameter exceeds 2 cm, when a 4:1 tunnel length-to-diameter ratio cannot be achieved, or when functional impairment is present.

7. Ureteroneocystostomy: Ureteroneocystostomy anastomosis is performed with 5-0 interrupted monofilament sutures after placing a double-J stent in the bladder (Figure 5).

8. Ureteral Reimplantation: The detrusor valves are closed over the ureter using a “top-down” approach. The first suture is placed at the top of the tunnel to stabilize the ureter (Figure 6), followed by a series of interrupted monofilament absorbable 4-0 sutures to complete the tunnel. A cotton tape is used to keep the ureter aligned and prevent it from bending during closure. Following the procedure, an abdominal drain is inserted through one of the 8 mm robotic trocar sites, and both a Foley catheter and a ureteral stent are left in place. The drain is removed before discharge if the output remains low.

### 2.3. Postoperative Management

All patients had an indwelling double-J stent left in place for 4 to 6 weeks postoperatively. All received antibiotic prophylaxis until the removal of the double-J stent. Renal and bladder USG was performed 1 month following the double-J stent removal and repeated 3 months later if the initial results were abnormal and thereafter once a year. Diuretic renogram was prescribed 1 year postoperatively in all patients.

### 2.4. Parameters Assessed

Preoperative parameters assessed were patients’ demographics, radiologic findings, presence of anatomical characteristics such as PUD, ectopic megaureter, and prior surgery on the same side, including endoscopic injection of bulking agent, endoscopic pneumatic balloon dilation, and prior transvesical ureteral reimplantation. 

Perioperative parameters evaluated were operative time (OT), length of hospital stay (LOS), use of analgesics, intra- and postoperative complications, postoperative febrile urinary tract infections (fUTIs), and follow-up duration. OT was measured from the placement of sterile drapes to the closure of port sites. Surgical success was defined as stable or improving HUN on USG indicated by a stable or reduced anteroposterior (AP) diameter, absence of symptoms, and improved washout parameters on diuretic renogram. Postoperative complications were assessed over a 30-day postoperative period and graded using the Clavien-Dindo classification [23].

### 2.5. Statistical Analysis

Statistical analysis was performed on SPSS Statistics version 23.0 (IBM Corp., Released 2015. IBM SPSS Statistics for Windows, Version 23.0. Armonk, NY, USA: IBM Corp.). 

Continuous and discrete variables are presented as medians and interquartile range (IQR). Categorical variables are reported as frequencies and percentages.

## 3. Results

A total of 16 patients with a diagnosis of complex obstructed megaureter underwent REVUR during the study period and were enrolled. Most of the patients (15/16, 93.7%) were male, and the median age at the time of surgery was 7.8 years (IQR: 2–16.3 years). Pathology was left-sided in the majority (10/16, 62.5%). 

COM was associated with paraureteral diverticula (*n* = 5), previous failed endoscopic balloon dilation (*n* = 4), prior transvesical ureteral reimplantation (*n* = 1), ectopic megaureter (*n* = 2), and previous endoscopic injection of bulking agent causing iatrogenic ureteral obstruction (*n* = 4). 

Presentation symptoms included breakthrough fUTIs (*n* = 8), flank pain (*n* = 4), hematuria (*n* = 2), and pseudo-incontinence (*n* = 2). 

The median preoperative ureteral diameter on the affected side was 1.77 cm (IQR 0.9–3.2 cm). Preoperative functional imaging was performed in all patients (100%), which included magnetic resonance urography, Mag3, or DMSA functional imaging, or a combination of these. The median preoperative split function of the affected kidney was 32.5% (IQR: 28.8–47.5). All patients had a T1/2 time for radiotracer washout greater than 20 min. Fourteen patients underwent preoperative voiding cystourethrogram (VCUG), showing preoperative VUR in three patients (21.4%). VUR was on the affected side in two patients and bilateral in one patient. 

All surgical procedures were accomplished robotically, with no need for conversions. No intra-operative complications occurred. Ureteral tapering was performed in 7/16 (43.7%). The median operative time (including robot docking) was 220 min (IQR: 155–290 min). The median docking time was 14.3 min (IQR: 11–22 min), and the median console time was 166 min (IQR: 135–211 min). Patients’ baseline and intraoperative details are summarized in Table 1.

The median length of stay (LOS) was 3.8 days (IQR: 3.2–7.0). The indwelling double J stent was removed 4–6 weeks postoperatively. At a median follow-up of 34.5 months (IQR: 6–58 months), none of the patients required reoperation for persistent obstructive megaureter. Clavien 2 grade complications occurred in 2/16 (12.5%) within 30 days postoperatively and included febrile UTI treated with oral antibiotics. 

All patients showed improvement in the degree of HUN on postoperative renal and bladder USG, as indicated by decreased ureteral diameter of the affected side. Postoperative renogram was obtained in 7/16 (43.7%) and showed a T1/2 time for radiotracer washout lower than 10 min. The median postoperative split function of the affected kidney was 32.5% (IQR: 14.8–47.5).

Three patients (two males, one female) developed postoperative febrile UTIs, with the median time from surgery to the first fUTI being 9.2 months (IQR: 6.5–13 months). 

Postoperative VCUG revealed evidence of Grade V reflux on the operated side in one female who presented fUTIs. She underwent an endoscopic subureteral injection of a dextranomer/hyaluronic acid (Dx/HA) bulking agent, with no further recurrence of fUTIs. Postoperative outcomes are summarized in Table 2.

## 4. Discussion

This paper reports on our recent experience with REVUR for treating obstructed megaureter in complex scenarios. While non-dismembered REVUR has been established as a viable alternative to open surgery in patients with primary VUR [6,12], there is limited experience about the role of REVUR for treating more complex cases, including POM [17,18,19]. Defining surgical success in this patient group can be challenging. The primary objective of surgery in patients with POM is to ensure adequate drainage and prevent further renal deterioration. Our findings confirm that REVUR is a highly effective and safe procedure, with significant reduction in ureteral dilation and improvement in symptoms and drainage. Complication rates were low, and no patients required reoperation for persistent obstruction within the follow-up period.

Our findings align well with the broader body of literature on robot-assisted surgery in pediatric urology, reinforcing the idea that robotic surgery offers precise dissection and reconstruction while minimizing morbidity, and REVUR is a strong option for complex ureteral conditions [17,18,19]. Like the open technique, completely dismembering the ureter from the bladder and removing the stenotic segment is crucial for the success of the procedure [24]. In the case of PUD, it is important to isolate and ligate the diverticulum as distally as possible from the bladder base before division. In the case of previous endoscopic balloon dilation or endoscopic injection of bulking agent, we did not encounter any challenges. In some cases, after ureteral dismembering, we were able to identify and remove the blebs of the injected bulking agent before reimplantation. Patients with previous transvesical surgery posed challenges. Robotic assistance allowed for precise control during dissection and suturing, which contributed to the reduction in postoperative complications [10].

Furthermore, the 2024 EAU/ESPU pediatric guidelines for pediatric VUR emphasize the role of minimally invasive techniques in managing complex ureteral conditions. Our results are consistent with these guidelines, which advocate for robotic approaches in cases where anatomy and pathology warrant such precision and reflect the growing consensus that robotic surgery is becoming the standard of care for challenging urological conditions in children [7].

The current study’s success rate and safety profile are in line with the findings of the largest robotic reimplant series for POM published until now by Mittal et al. in 2021 [25]. They highlighted the advantages of robotic systems, such as enhanced visualization and dexterity, which were the key factors for such positive outcomes. They separately analyzed operative time for tapered vs. non-tapered REVUR and reported a significant difference between the two groups, with a mean time of 331.3 ± 76.9 min in the tapered cohort vs. 230.3 ± 39.9 min. Our operative time, including both tapered and non-tapered cases, was significantly shorter than that reported by Mittal et al. [25] and by Li et al. [24]. This was probably due to previous experience with simple REVUR that we developed before approaching such a more challenging procedure.

Our study’s follow-up data revealed that no patients required reoperation for persistent obstruction, and all showed improvement in hydronephrosis. This outcome mirrors the long-term success rates reported by Baek and Koh in 2017 [13], who emphasized that robotic surgery leads to durable outcomes over time. Their decade-long experience with pediatric robotic ureteral reimplantation demonstrated that careful patient selection and surgical expertise are essential for achieving these positive results. Other studies [17,18,19] also reported favorable outcomes for robotic-assisted reimplantation after failed endoscopic treatments for VUR, indicating that the robotic approach can effectively address even the most complex urological anomalies. Our findings strongly support that robotic surgery should be considered not only as a primary intervention but also as a salvage option in cases where other treatments have failed.

In the pediatric literature, there is a small amount of data regarding the long-term follow-up of pediatric patients receiving robotic ureteral reimplantation to treat POM. Most studies have reported success rates up to 100% in resolving obstruction [25,26]. Long-term follow-up revealed that most patients sustained resolution of the obstruction over time, with no recurrence of symptoms or the need for further interventions. One of the main goals in treating obstructive megaureters is to preserve renal function. A long-term follow-up study [26] showed that robotic ureteral reimplantation can prevent further deterioration of kidney function. The authors reported a significant reduction in hydronephrosis on follow-up ultrasounds and stable kidney function in terms of serum creatinine level and glomerular filtration rate. They reported a low incidence of postoperative complications, mostly grade I and grade II Clavien–Dindo. No patients required reoperation for recurrent ureteral obstruction. Future larger studies with additional follow-up are needed to determine the efficacy of the robotic approach to addressing POM.

The role of ureteral tapering in the surgical repair of POM remains uncertain. Mittal et al. [25] did not demonstrate a clear advantage in reducing the risk of postoperative febrile UTIs with or without tapering. The criteria to determine the need for ureteral tapering are typically based on anatomical and functional factors, particularly related to the size and function of the ureter. Tapering is often recommended if the ureter remains excessively dilated after decompression [4]. A frequently cited cutoff is a diameter greater than 1.5 cm. The ureter’s size is assessed intraoperatively, and tapering is performed if the dilatation is significant enough to impair normal function or prevent effective reimplantation. In reconstructive surgeries like ureteral reimplantation, an adequate ratio between the submucosal tunnel length and ureteral diameter is essential to prevent complications like reflux. A common benchmark is a 4:1 ratio (tunnel length to ureteral diameter). If the ureter is too large to achieve this ratio, tapering may be necessary. A recent concept suggests that the full 5:1 tunnel-length-to-ureteral-diameter ratio, as originally proposed by Paquin, may not be necessary. Achieving this ratio can be difficult, particularly in cases of megaureter and small bladder sizes. A more flexible approach allowing for a smaller ratio can reduce or eliminate the need for tapering or a long tunnel. Babu [27] introduced a “mini reimplantation” technique for the megaureter, avoiding tapering during intravesical reimplantation. In his series of 13 patients, he created a 2:1 tunnel ratio, with only two cases of postoperative reflux, with comparable outcomes to 15 patients who underwent classic Cohen reimplantation with excisional tapering. However, the tapered cases had a higher incidence of obstruction and greater need for reoperation. Similarly, Villanueva [28] presented a study of nine infants under six months old needing surgery for obstructive megaureter. Instead of cutaneous ureterostomy, he performed a “mini” extravesical reimplantation using a 2–3 cm tunnel, regardless of ureteral diameter. In the last five patients, he applied “mini-tapering” over the distal 2–3 cm of the ureter. While two of the first four patients experienced postoperative reflux, none of the last five had clinically significant vesicoureteral reflux (VUR) during a median follow-up of 44 months. In cases with functional obstruction, where the ureter is dilated and peristalsis is impaired or obstructed, tapering may be needed to restore proper function. Non-functioning segments of the ureter may be identified through imaging or intraoperative assessment, guiding the decision to taper. Even after dismembering or decompression, if the ureter remains significantly dilated, tapering may be considered to facilitate proper flow and avoid postoperative complications. 

In our experience, the decision for ureteral tapering is approached intraoperatively with great caution. After dismembering, the ureteral diameter is measured, and we generally proceed with tapering only if the ureteral diameter exceeds 2 cm, when it hinders the creation of an effective anti-reflux tunnel, or when functional impairment is present. However, since ureteral diameter measurements during laparoscopic procedures are often based on subjective assessments, the decision to taper is influenced more by the surgeon’s preference than by a strict size criterion. We tend to favor ureteral tapering, particularly in female patients, due to their higher risk of developing febrile UTIs. The technique for robot-assisted intracorporeal ureteral tapering is still evolving. We prefer to perform the excisional tapering after disarticulating the ureter from the UVJ. We initially put the first sutures of ureteroneocystotomy to maintain tension. Thereafter, we taper and suture the ureter with simple monofilament sutures.

While our study provides valuable insights into the efficacy and safety of REVUR for treating complex POM, several limitations should be considered. The retrospective nature of this study inherently limits the ability to establish causality. Although we utilized an IRB-approved robotic surgery registry to identify patients and minimize bias, retrospective studies are still susceptible to selection bias and incomplete data. 

This study was conducted at a single institution, which limits the generalizability of the findings. Although the results are promising, the relatively small sample size, with only 18 patients enrolled, limits the statistical power of the study. Larger studies are necessary to confirm these findings and identify potential risk factors for adverse outcomes.

## 5. Conclusions

This study demonstrates that robot-assisted extravesical ureteral reimplantation was a safe and effective treatment for primary obstructive megaureter and other complex ureteral anomalies in our patient cohort. The procedure showed low complication rates, high success rates, and favorable long-term outcomes, supporting the feasibility and effectiveness of robotic surgery for these conditions. Future prospective, multi-center studies with larger sample sizes and standardized protocols will be essential to further confirm the role of robotic surgery in managing primary obstructive megaureter in children.

## Figures and Tables

**Table 1:** Patients’ baseline and intraoperative details.

Total Patients, *n*	16	
Male, *n* (%)	15	93.7%
Female, *n* (%)	1	6.3%
Age at surgery (years), median (IQR)	7.8	(2–16.3)
Laterality:		
Left, *n* (%)	10	62.5%
Right, *n* (%)	6	37.5%
Bilateral, *n* (%)	0	0%
Complex Anatomy:		
Paraureteral Diverticulum (PUD), *n* (%)	5	31.2%
Ectopic Megaureter, *n* (%)	2	12.5%
Previous surgery, *n* (%)	9	56.2%
Clinical Presentation:		
Breakthrough febrile UTIs	8	50%
Flank Pain	4	25%
Gross Hematuria	2	12.5%
Pseudo-incontinence	2	12.5%
Pre-op Ureteral Diameter Affected Side (cm), median (IQR)	1.77	(0.9–3.2)
Ipsilateral Renal Function (%), median (IQR)	32.5	(28.8–47.5)
Pre-operative VUR on the affected side, *n* (%)	3	18.7%
Operative time (minutes), median (IQR)	220	(155–290)
Docking time (minutes), median (IQR)	14.3	(11–22)
Console time (minutes), median (IQR)	166	(135–211)
Conversion, *n* (%)	0	0%
Intraoperative complications, *n* (%)	0	0%
Ureteral Tapering, *n* (%)	7	43.7%

**Table 2:** Postoperative outcomes.

Total Patients, *n*	16	
Length of stay (days), median (IQR)	3.8	(3.2–7.0)
Clavien–Dindo complications < 30 days after surgery		
Total number, *n* (%)	2	12.5%
Grade II, *n* (%)	2	12.5%
Clavien–Dindo complications > 30 days after surgery		
Total number, *n* (%)	3	18.7%
Grade II, *n* (%)	3	18.7%
Total follow-up (months), median (IQR)	34.5	(6–58)
Postop Ureteral Diameter Affected Side (cm), median (IQR)	0.7	(0.4–0.9)
Postop Ipsilateral Renal Function (%), median (IQR)	32.5	(14.8–47.5)
Postop VUR on the affected side, *n* (%)	1	(6.2%)
Re-operation, *n* (%)	1	(6.2%)

## Data Availability

The data presented in this study are available on request from the corresponding author due to privacy reason.
